# Nonnative implicit phonetic training in multiple reverberant environments

**DOI:** 10.3758/s13414-019-01680-0

**Published:** 2019-02-08

**Authors:** Eleni Vlahou, Aaron R. Seitz, Norbert Kopčo

**Affiliations:** 1 Department of Psychology, University of California, Riverside, 900 University Avenue, Riverside, CA 92521 USA; 2 Institute of Computer Science, Faculty of Science, P. J. Šafárik University, Košice, Slovakia; 3 Hearing Research Center and Department of Biomedical Engineering, Boston University, Boston, MA USA

**Keywords:** Adaptation to reverberation, Speech perception, Phonetic learning, Implicit learning, Perceptual learning

## Abstract

Speech intelligibility is adversely affected by reverberation, particularly when listening to a foreign language. However, little is known about how phonetic learning is affected by room acoustics. This study investigated how room reverberation impacts the acquisition of novel phonetic categories during implicit training in virtual environments. Listeners were trained to distinguish a difficult nonnative dental-retroflex contrast in phonemes presented either in a fixed room (anechoic or reverberant) or in multiple anechoic and reverberant spaces typical of everyday listening. Training employed a videogame in which phonetic stimuli were paired with rewards delivered upon successful task performance, in accordance with the task-irrelevant perceptual learning paradigm. Before and after training, participants were tested using familiar and unfamiliar speech tokens, speakers, and rooms. Implicit training performed in multiple rooms induced learning, while training in a single environment did not. The multiple-room training improvement generalized to untrained rooms and tokens, but not to untrained voices. These results show that, following implicit training, nonnative listeners can overcome the detrimental effects of reverberation and that exposure to sounds in multiple reverberant environments during training enhances implicit phonetic learning rather than disrupting it.

The role of reverberation in auditory perceptual learning has received little attention, even though reverberation is a ubiquitous component of stimuli that we hear in natural environments. Evidence suggests that reverberation can have a negative effect, impairing speech perception (Devore & Shinn-Cunningham, 2003; Nábĕlek & Donahue, 1984; Takata & Nábĕlek, 1990; Ueno et al., 2005) or sound localization in the horizontal plane (Hartmann, 1983). On the other hand, reverberation can be beneficial—for example, serving as a cue for distance perception (Zahorik, Brungart, & Bronkhorst, 2005) and stream segregation (David, Lavandier, & Grimault, 2014). While the auditory system has mechanisms to adapt to reverberant environments and to mitigate some of the negative impacts of reverberation (for speech, see Beeston et al., 2014; Brandewie & Zahorik, 2010; Srinivasan & Zahorik, 2013; Watkins, 2005; for distance, Shinn-Cunningham, 2000), it is clear that reverberation has a substantial impact on auditory perception and a likely impact on our ability to learn to discriminate different sound types and to generalize this information to novel acoustic environments.

Although there is a limited understanding of how listeners are affected by reverberation in the process of learning novel phonetic categories, important findings can be drawn from research on nonnative speech perception in complex listening settings. These studies show that adults face persistent difficulties when processing nonnative speech in adverse conditions, such as background noise, competing talkers, time-compressed speech, and cognitive load, even after years of experience with the nonnative language (Banai & Lavner, 2016; Cooke, Garcia Lecumberri, & Barker, 2008; Lecumberri, Cooke, & Cutler, 2010; Rogers, Lister, Febo, Besing, & Abrams, 2006). For example, nonnative listeners are more adversely affected by noise when the task involves the perception of words and sentences (see Lecumberri et al., 2010, for a review). Rapid adaptation to time-compressed speech is weaker in a nonnative language, and, even after intensive training, nonnative listeners still perform more poorly compared with naïve native listeners when tested with novel sentences (Banai & Lavner, 2016), whereas interference from competing speech maskers is stronger (Cooke et al., 2008). Collectively, these results suggest that various sources of speech degradation are likely to interfere with nonnative perceptual learning. Here, we focus on the less investigated aspect of room variation on the perceptual learning of a nonnative phonetic distinction.

A child learning its first language or an adult learning a new language must learn new phonetic categories in conditions in which the phonetic stimuli are distorted by reverberation. Reverberation is likely to affect phonetic learning in particular in adulthood, as adults’ learning of sounds of a new language can be very challenging even under optimal listening conditions. This is because foreign speech is filtered through the native phonological system, imposing severe constraints on how we perceive certain nonnative phoneme distinctions (Flege, 2003; Iverson et al., 2003). Under adverse conditions, reverberation can act as “noise,” further impeding the perception of acoustic differences to which listeners are already mistuned due to extensive experience with their native language. Compared with native listeners, nonnative listeners are more adversely affected by reverberation when listening to speech (Nábĕlek & Donahue, 1984; Takata & Nábĕlek, 1990; see also Lecumberri et al., 2010, for a review), and this difference becomes more pronounced for rooms with longer reverberation times (Nábĕlek & Donahue, 1984; Peng & Wang, 2016). However, little is known about how increased difficulty in speech processing affects phonetic learning in rooms.

The main question examined here is whether exposure to multiple acoustic environments during training is beneficial or detrimental to phonetic learning. On the one hand, introducing varying levels of distortions to the stimuli might make the phonetic learning task harder. On the other hand, this variability might facilitate listeners’ perception of the new sounds across different listening environments. For example, research on word learning in adverse conditions suggests that listeners’ representations of the newly learned material retain aspects of the acoustic environment that may not be optimal in different listening settings (Creel, Aslin, & Tanenhaus, 2012). Exposure to different acoustic environments during training might help overcome such specificities, allowing listeners to identify the phonetic cues that are more resistant to distortions caused by room acoustics and learn to adapt to different critical cues in different listening environments. Here, we examine whether exposure to a single or to multiple acoustic environments enhances or impedes perceptual learning of nonnative phonetic contrasts.

Previous studies showed significant adult phonetic learning for various nonnative sounds, though performance typically does not reach native levels and there is substantial individual variability in learning (Flege, 2003; Holt & Lotto, 2010; Strange & Dittmann, 1984; Takagi, 2002). A consistent finding in the phonetic learning literature is that ample variability during training along various acoustic-phonetic dimensions, such as different phonetic contexts and, importantly, multiple voices, is necessary to form robust phonetic categories and to overcome overspecified, talker-specific representations (e.g., Lively, Logan, & Pisoni, 1993; Logan, Lively, & Pisoni, 1991; Pruitt, Jenkins, & Strange, 2006). Furthermore, feedback during training is an influential factor in learning, guiding listeners’ attention to phonetic properties that reliably differentiate target phonemes (Logan et al., 1991). Participants are typically provided with feedback on the correctness of their responses on a trial-by-trial basis (Golestani & Zatorre, 2004; Iverson, Hazan, & Bannister, 2005; Lively et al., 1993; Logan et al., 1991; Pruitt et al., 2006). However, although explicit performance feedback is beneficial, it is rare in naturalistic environments. Accordingly, alternative designs have investigated unsupervised and implicit learning via internally generated reward signals in lieu of feedback (Lim & Holt, 2011; Vlahou, Protopapas, & Seitz, 2012).

In particular, the task-irrelevant perceptual learning (TIPL) framework posits that, when participants are engaged in a task, important stimuli and events that are successfully processed elicit diffuse release of neuromodulatory signals that gate plasticity (Seitz & Watanabe, 2003, 2005, 2009). Thus, at the time of important task events (such as when a task target is discovered), learning may take place for other stimuli, whether or not they are relevant or attended, as long as they are systematically paired with those task events. This model has been applied to learning of speech components and nonnative speech sounds and, in some cases, has produced equal or greater performance benefits than is found through explicit training (Seitz et al., 2010; Vlahou et al., 2012). The current study focuses on implicit learning. Specifically, we employ a variant of TIPL in which phonetic stimuli coincide with rewards, but do not improve performance in a central task, to test the hypothesis that concurrence of stimulus processing and task-related reward signals is sufficient for learning, even when the stimulus has no predictive value for the task.

We present the results of a behavioral experiment that examined implicit phonetic training in varying acoustic environments. We trained adult listeners on nonnative speech sounds in simulated anechoic and reverberant rooms. We used the well-known Hindi dental/retroflex contrast, a phonetic distinction that is used in approximately 11% of the world’s languages (Golestani & Zatorre). Several studies show that adults find it difficult to differentiate the phonemes in standard discrimination and categorization tests, but are able to improve after laboratory training (Golestani & Zatorre, 2004; Vlahou et al., 2012; Werker & Tees, 1984). Five different rooms were simulated to capture a range of everyday environments in which a listener is typically exposed to speech: an office, a classroom, a cafeteria, a bathroom, and an anechoic room, approximating an outdoor environment with no acoustic reflections. Different groups of participants were trained in a single acoustic environment (anechoic or reverberant) and in multiple rooms (anechoic and two reverberant rooms) with speech sounds produced by a single Hindi voice. Before and after training, they were tested in trained and untrained rooms, with trained and untrained speech tokens produced by the Hindi voice used during training, and with speech tokens produced by an unfamiliar Hindi voice. We focused on implicit learning for two reasons. First, the effect of explicit training on phonetic learning is well established and robust. Second, it can be expected that the effect of room variation is particularly important for implicit learning, as the room-to-room variation can be critical for automatic bottom-up identification of the room-invariant features that can be used to distinguish the phonemes in various reverberant environments. For training, we used a TIPL videogame in which phonetic stimuli from one category were consistently paired with rewards in the game, without contributing to performance improvement.

Our main research questions were as follows: First, to test whether the implicit training paradigm would induce perceptual learning on the nonnative phonetic categorization task across rooms with varying levels of reverberation. We expected that trained participants would outperform untrained listeners from a control condition. Second, to investigate differences in the magnitude of learning between the two training conditions (one fixed room vs. three varying rooms), although we were agnostic as to whether room variation during training would be beneficial or detrimental to phonetic learning. Finally, to examine transfer of learning at posttesting to untrained material (tokens, rooms, voices). We expected improved performance across untrained speech tokens from the trained speaker, and limited or no learning for the untrained speaker. This prediction was based on our earlier work (Vlahou et al., 2012) and on research on the effects of variability in nonnative phonetic learning (e.g., Lively et al., 1993; Pruitt et al., 2006), which suggests that generalization of learning occurs only for dimensions in which there is ample variability during training. Based on the same logic, the strongest candidate to show generalization of learning across untrained rooms was the three-varying-rooms condition, in which participants were exposed to varying room environments during training.

## Method

### Participants and conditions

Forty-two adults (26 females, 16 males, 18−37 years old) participated in the experiment. Each participant was assigned to one experimental group. The groups differed by the type of training that participants underwent, referred to as the experimental condition. Table 1 summarizes the experimental conditions and the number of participants per condition. Thirteen individuals participated in a no-training control condition consisting of pretest and posttest without any training. Twenty-nine participants received implicit training. Fifteen of them received training using sounds from three rooms (anechoic and two reverberant rooms, bathroom and classroom, described below; 3R condition). Fourteen participants received training in a single room (1R condition), either anechoic (eight participants) or reverberant (six participants—four trained in the classroom and two in the bathroom). One participant from the 1R condition was dropped from the study due to missing data, and thus only 13 participants were included for this condition in the analyses reported below. Most participants were native speakers of English. Two of them were bilingual, with Spanish (1R) or Tagalog (3R) as a second language. Two participants were native speakers of Spanish (one in 1R and one in 3R), one of Slovak (3R) and one of Vietnamese (3R). No participant reported any hearing impairments or any experience with the Hindi language. Participants gave written informed consent approved by the University of California at Riverside Human Research Review Board and were compensated $10 for each experimental session that they completed.

**Table 1:** Experimental conditions and number of participants per condition

Condition	Rooms used during training	Number of participants
Control	None	13
3R	3 rooms (anechoic, bathroom and classroom)	15
1R	1 room (anechoic or bathroom or classroom)	13 (7 in anechoic, 2 in bathroom, 4 in classroom)

### Stimuli and setup

The stimuli were natural recordings of Hindi syllables from two male native Hindi speakers. We used the Hindi dental-retroflex voiceless stop followed by the long [i:] (for further details on the recordings and processing of the sounds, see Vlahou et al., 2012). There were in total 20 different tokens from each Hindi speaker, 10 beginning with a dental sound and 10 with a retroflex sound.

Five different rooms were simulated—an office, a classroom, a cafeteria, a bathroom, and an anechoic room. Each original speech syllable was convolved with the left-ear and right-ear binaural room impulse responses (BRIRs) recorded in each of the rooms at a location directly in front of the listener. This resulted in five different versions of each original syllable, one per simulated room. A detailed description of the rooms and BRIR recording procedures for the classroom and bathroom BRIRs is provided in Shinn-Cunningham, Kopčo, and Martin (2005) and Devore and Shinn-Cunningham (2003), respectively. A description of the office and cafeteria BRIRs is provided in Kayser et al. (2009). The anechoic impulse response was derived from the classroom BRIR by removing the reverberant portion by applying a 1-ms linear ramp just prior to the onset of the first reflection. Basic acoustic parameters, T_60_ and C_50_, of the rooms are summarized in Table 2. These values were computed by only considering the first 300 ms of each BRIR (to prevent noise floor from influencing the values), using the impulse response acoustic information calculator (Version 1.5.4; https://www.mathworks.com/matlabcentral/fileexchange/42566-impulse-response-acoustic-information-calculator) in MATLAB (The MathWorks Inc., Natick, MA). All stimuli were high-pass-filtered at 70 Hz and presented at the sampling rate of 44.1 kHz from an Apple G5 computer Burr Brown PCM3052 sound card via Senheiser 650 headphones, at a comfortable level individually adjusted for each participant.

**Table 2:** Acoustic parameters T_60_ and C_50_ of the left channel binaural room impulse responses for each simulated room

Room	Broadband T_60_ (ms)	Broadband C_50_ (dB)
Bathroom (BA)	658	18.5
Cafeteria (CA)	605	57.7
Classroom (CL)	528	34.1
Office (OF)	348	22.4
Anechoic (AN)	~0	∞

### Procedure

The experiment consisted of three phases: pretraining testing (pretest), training, and posttraining testing (posttest), all conducted in a sound-proof laboratory at the University of California, Riverside. For testing, stimulus presentation was controlled by DMDX scripts (Forster & Forster, 2003). For the implicit training, we used a videogame written in Python (http://www.python.org/) using Pygame (http://www.pygame.org/).

#### Testing

Two identical testing sessions were performed: a pretest on a day before the first training session and a posttest a day after the last training session (if the testing day fell on a weekend day, it was shifted to the nearest business day). Prior to testing, participants were informed of the Hindi phonetic categories (termed “T1” and “T2”; for a half of the participants, “T1” was associated with retroflexes and for the other half with dentals). During testing on stimuli from one speaker, that speaker’s speech tokens convolved with the BRIRs presented in five blocks of 40 trials, one block for each room. Each 40-trial block consisted of two presentations of the speaker’s 20 tokens (10 per phoneme) in a random order.

For each room and speaker, prior to the first trial of each pretest block, there was a brief familiarization phase where listeners were first informed about which room was about to be tested, and then listened to two identical practice sequences, each consisting of five different pairs of tokens (each pair containing one dental and one retroflex token) while seeing the label T1 or T2 on the screen. Participants were given feedback during familiarization, but not during subsequent tests.

**Fig. 1:**
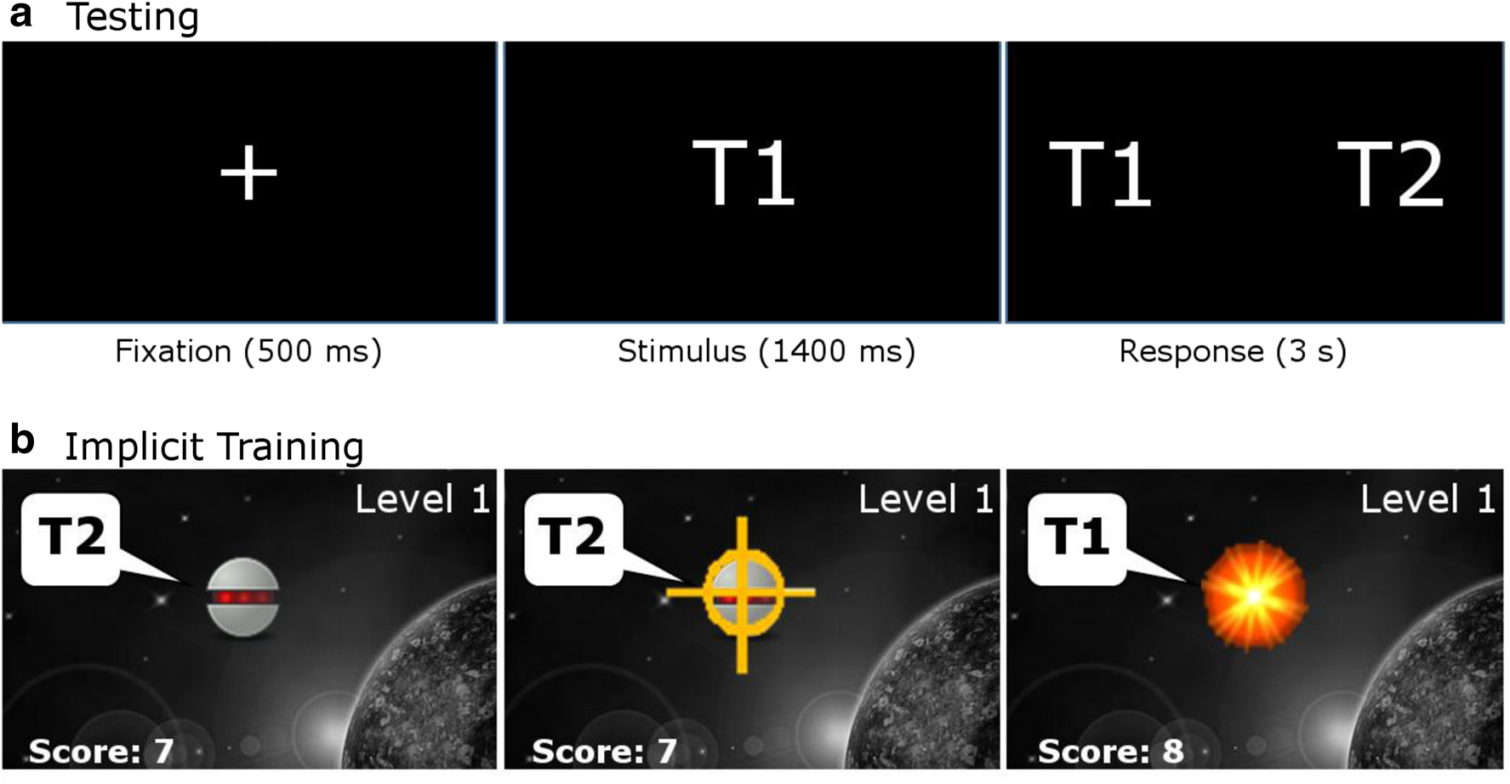
Task schematics. **a** Testing. Schematic of the one interval, two-alternative forced-choice phonetic categorization task; after the trial initialization, participants were presented with one sound token from one of the categories and were required to report whether it belonged to T1 or T2. No feedback was provided during testing. **b** Implicit training. Schematic of the videogame; a moving target appeared at a random screen location (left-hand panel), producing two sounds from the “background” category (T2). Participants were required to aim and shoot the alien character (center panel). If the player succeeded, an explosion appeared and two sound tokens from the “target” category (T1) were presented (right-hand panel)

The two Hindi speakers were tested in separate blocks, with the speaker used during training (see the Training subsection) always tested first. The testing procedure was a standard one-interval, two-alternative forced-choice phonetic categorization task (see Fig. 1a). Throughout testing, the labels “T1” and “T2” were shown on the screen. In each trial, one syllable was presented half of the time with the dental and half with the retroflex one. Then a message “Was the sound from T1 or T2?” appeared. Participants had to identify the category to which the sound belonged by pressing the key assigned to each label. If a participant did not respond within 3 s after the sound presentation, the response was considered incorrect. Each testing session consisted of 400 trials (all combinations of 10 tokens, two repetitions, two phonemes, two speakers, and five rooms) and lasted approximately 45 minutes.

#### Training

The training stimulus set consisted of five tokens of each phoneme (fixed across participants) spoken by one of the Hindi speakers. The speaker was randomly selected and counterbalanced across participants. Training was usually conducted over a period of 5 days, and no more than 10 days. Each daily session consisted of 600 trials. In the 1R training condition, all 600 trials came from a single room (classroom, bathroom, or anechoic). In the 3R training condition, 200 trials per room were performed in the classroom, bathroom, and anechoic room. Each session consisted of 40-trial blocks in which the room was fixed. Five such blocks were performed per room, with the room randomly changing from one block to the next. Phonetic tokens within each block were randomly ordered.

Training (see Fig. 1b) was in the form of a videogame. On each trial, a moving character appeared at a random location on the screen (initial motion speed of approximately 2°/s). The participant’s task was to aim and shoot the character as fast as possible. Three consecutive hits resulted in a small increase in the character’s motion speed on the following trial by approximately 1°/s, whereas each miss resulted in an equal decrease in speed, with a minimum speed of 2°/s. If a participant failed to produce a response within 2 s after the character was presented, the trial ended and was treated as a miss. Once the participant’s performance reached a predefined threshold, the game moved to a higher level (up to the highest, fifth level), with the only difference being a different graphic environment and increased initial speed. Throughout the procedure, performance statistics (number of targets eliminated, level) appeared on the screen, as a motivational feature. Upon character appearance, a randomly selected token sound from one phonetic category was presented twice in a succession without any pause. This sound represented the “background” (counterbalanced, so that for one half of the participants the background sound was dental and for the other half retroflex). If the participant succeeded in shooting the moving character, the score increased by 1 point, an explosion appeared on the screen for approximately 150 ms, and a randomly selected token from the “target” category (explained below) was presented twice in a succession (no sound was presented in case of a miss).

The important experimental manipulation consisted of pairing sounds from one category (“target”) with proper reinforcement schedules in the game. Specifically, TIPL postulates that successful task performance (here, hitting the visual character) triggers release of reinforcement learning signals that drive plasticity. These signals can be triggered via internal processes (i.e., participants monitoring their own success) and external rewards (i.e., game features such as the explosion and the increase in score). Importantly, these learning signals are nonspecific to the events that caused their elicitation and can result in learning of unrelated stimuli that temporally coincide (Seitz & Watanabe, 2005, 2009). Thus, by systematically presenting sounds from the “target” category that temporally coincide with times of reinforcement, the critical acoustic features of the speech tokens stand out and can be strengthened and refined.

### Data analysis

Test performance for all conditions was assessed by computing percentage correct identification of the sound phonetic category, using the T1 and T2 labels, which were randomly assigned to dental and retroflex stimuli on a per-participant basis. The proportion correct data were logit-transformed and entered into repeated-measures ANOVA analyses, using the Greenhouse–Geisser correction where appropriate. Unless specified differently, data figures show across-participant means and error bars are standard errors of the means, corrected for within-participants designs (Cousineau, 2005; Morey, 2008). Improvement in performance during training was assessed by evaluating the change in average speed (°/s) across training sessions. This measure is an indirect indicator of performance because as participants became more skilled at hitting the moving targets, the targets’ motion speed increased.

## Results

### Effect of training

**Fig. 2:**
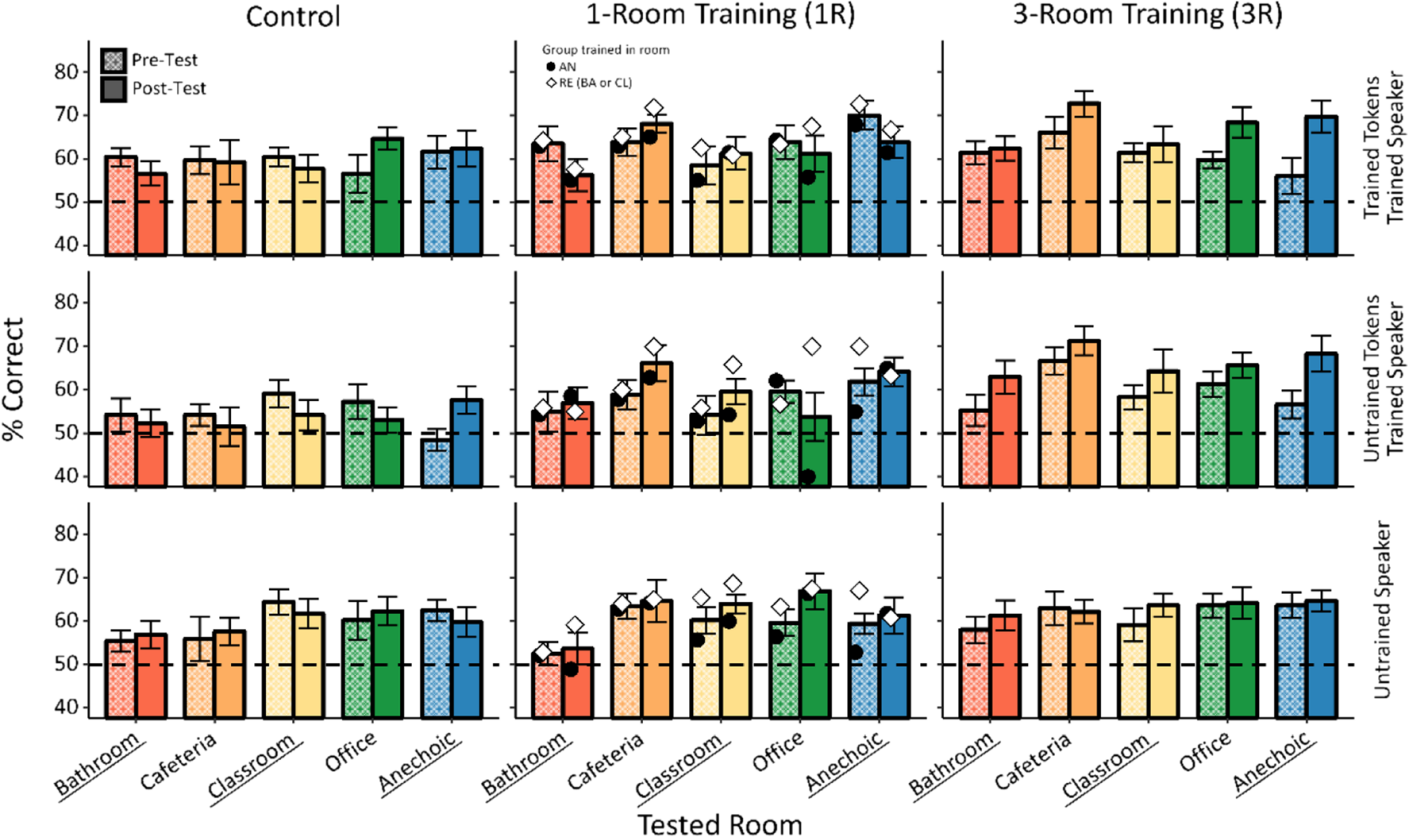
Testing performance. Consonant identification accuracy at pretest (lighter bars) and posttest (darker bars) as a function of testing room for trained tokens (top row), untrained tokens (middle row) and the untrained speaker (bottom row). Rooms used during training for each condition are underscored (note that for the 1R condition, each participant was trained in only one room). Symbols in the 1R panels show performance separately for the groups trained in the anechoic room (AN) and reverberant rooms (RE). Horizontal dashed line indicates chance performance

Our first research goal was to test the effectiveness of the current implicit training paradigm in improving listeners’ performance in the phonetic categorization task. We hypothesized that trained listeners would outperform untrained participants at posttesting. Figure 2 presents pretest and posttest performance for the different conditions (left-hand vs. central vs. right-hand column) as a function of testing room, separately for the trained and untrained speech tokens from the trained speaker (top and middle row) and the speech tokens from untrained speaker (bottom row).

The results in this figure partially support our prediction. Specifically, focusing on performance for trained and untrained speech stimuli from the trained speaker, the results show that learning differed across conditions. Consistent with our hypothesis, participants in the three-room condition (3R) improved their performance at posttesting for both trained and untrained speech tokens, whereas no evidence of learning was observed for the control condition. On the other hand, contrary to our predictions, negligible improvements were observed for participants trained in the one-room (1R) condition, suggesting that the current implicit training paradigm in a fixed environment was ineffective. Finally, for the untrained speaker (bottom row), there was no evidence of learning in any of the conditions, consistent with our previous work and the literature (e.g., Lively et al., 1993; Pruitt et al., 2006; Vlahou et al., 2012).

These results were subjected to statistical testing. We first focused on performance on trained and untrained speech stimuli from the trained speaker, where the strongest learning effects were expected (see Vlahou et al., 2012). A mixed ANOVA with condition (1R, 3R, control) as a between-participants factor and with time (pretest, posttest), room (bathroom, cafeteria, office, classroom, anechoic), and token type (trained tokens, untrained tokens) as within-participants factors found significant main effects of token type, *F*(1, 38) = 5.45, *p* = .025, η_p_^2^ = 0.13; room, *F*(4, 152) = 3.87, *p* = .008, η_p_^2^ = 0.09; and time, *F*(1, 38) = 5.40, *p* = .0255, η_p_^2^ = 0.12; and Token Type × Time × Room, *F*(4, 152) = 2.76, *p* = .035, η_p_^2^ = 0.07. Importantly, there was a significant Time × Condition interaction, *F*(2, 38) = 3.93, *p* = .028, η_p_^2^ = 0.17, suggesting that there was learning for some conditions, but not for others. Furthermore, this learning was not limited to the particular speech tokens presented during training, as no interaction involving time, condition, and token type was significant (with the exception of the Token Type × Time × Room interaction, all interactions involving token type and time were nonsignificant; Token Type × Time: *F*(1, 38) < 1, *ns*; Condition × Token Type × Time: *F*(2, 38) < 1, *ns*; Condition × Token Type × Room × Time: *F*(8, 152) < 1, *ns*. Although the Token Type × Time × Room interaction might be of interest in other contexts, it was not further analyzed here, as the main focus of this study is on the type of training (condition). Instead, in the following analysis, the trained and untrained speech tokens from the trained speaker were pooled together.

To further investigate the significant Time × Condition interaction, separate ANOVAs were performed focusing on how the amount of learning varied across the different types of training (condition). We first examined performance in the control condition. Repeated measures (RM) ANOVA with factors of time, room, and speaker found no significant main effects or interactions, *F*(4*,* 48) = 1*.*82*, p* = *.*139 for room; *F*(1*,* 12) = 1*.*64*, p* = *.*224 for speaker (all other *F*s *<* 1). The lack of main effects or interactions involving time confirmed that repeated testing without training did not yield improvements in performance.

**Fig. 3:**
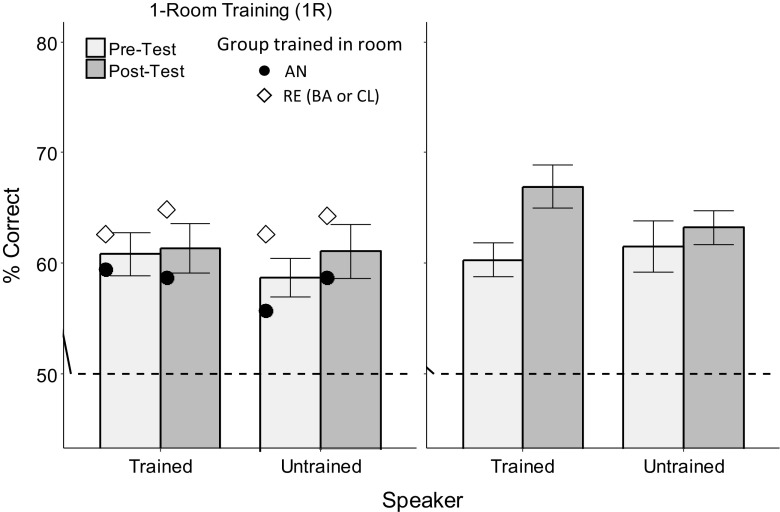
Performance at pretest and posttest plotted as a function of trained (tested first) and untrained speaker (tested second) for data collapsed across testing rooms. Symbols in the 1R panels show performance separately for the groups trained in the anechoic room (AN) and reverberant rooms (RE). Dashed line indicates chance performance

Our second main research question was to investigate the differences between the two implicit training conditions. Figure 3 presents pretest and posttest performance for the one-room and three-room implicit conditions. A mixed ANOVA, with condition (1R, 3R) as a between-participants factor and time, room, and speaker as within-participants factors, showed that performance varied across rooms (main effect of room), *F*(4, 104) = 8.02, *p* = .0002, η_p_^2^ = 0.24. More pertinent to our research questions, there was a significant improvement from pretest to posttest (main effect of time), *F*(1, 26) = 6.28, *p* = .019, η_p_^2^ = 0.19, interacting with condition and speaker, *F*(1, 26) = 4.43, *p* = .045, η_p_^2^ = 0.15, but not with room. The significant Time × Condition × Speaker interaction shows that the improvement was approximately evenly distributed across rooms, but differed depending on the number of rooms used in training and on whether the tested speaker differed from the trained speaker.

To further investigate this, Fig. 3 plots the data collapsed across rooms and separately for the two speakers and the different training conditions. This figure shows that there was no improvement due to 1R training either for the trained or the untrained speaker, but that there was an improvement in performance of approximately 7% due to 3R training for the trained speaker. However, this improvement did not generalize to the untrained speaker.

To confirm these observations, partial ANOVAs were performed separately for each type of training. For the 3R data (see Fig. 3, right-hand panel), a RM ANOVA, with speaker and time as factors, showed a significant main effect of time, *F*(1, 14) = 8.12, *p* = .013, η_p_^2^ = 0.37, and a significant Time × Speaker interaction, *F*(1, 14) = 7.48, *p* = .016, η_p_^2^ = 0.35. Paired *t* tests showed significant learning for the trained speaker, *t*(14) = −4.33, *p* = .0007, and no improvement for the untrained speaker, *t*(14) = −0.82, *p* = .43. This result shows that when implicit training is performed in three varying reverberant and anechoic rooms, participants can learn to discriminate the new nonnative phonetic contrast, while no such learning occurs when only one room is used during training.

Because many of the participants in the 1R group were trained only in the anechoic room, it is not clear whether the critical feature of the 3R training versus the 1R training was that reverberation was present during a majority of 3R training trials (whereas none was present for the 1R anechoic participants) or that the amount of reverberation varied a lot across the 3R training trials (whereas it was fixed for the 1R anechoic as well as reverberant participants). To distinguish these two options, a partial ANOVA was performed on the 1R training data in which participants were further split into two different groups, the anechoic 1R group (seven participants; circles in Fig. 3) and the reverberant 1R group (four participants in classroom, two in bathroom; diamonds in Fig. 3). A mixed ANOVA, with group (1R-anechoic, 1R-reverberant) as a between-participants factor and time and speaker as within-participants factors, found no difference between the two training groups, no improvement from pretest to posttest, and no interactions, condition, *F*(1, 12) = 1.98, *p* = .184 (all other *F*s < 1, *ns*). The result that there was no learning for either the 1R-AN or 1R-RE group suggests that the critical factor for the implicit learning paradigm for the 3R group was the room variation, as opposed to just presence of reverberation. However, due to the small number of participants in the 1R subgroups, the results of this analysis should be examined with caution.

### Generalization of learning

**Fig. 4:**
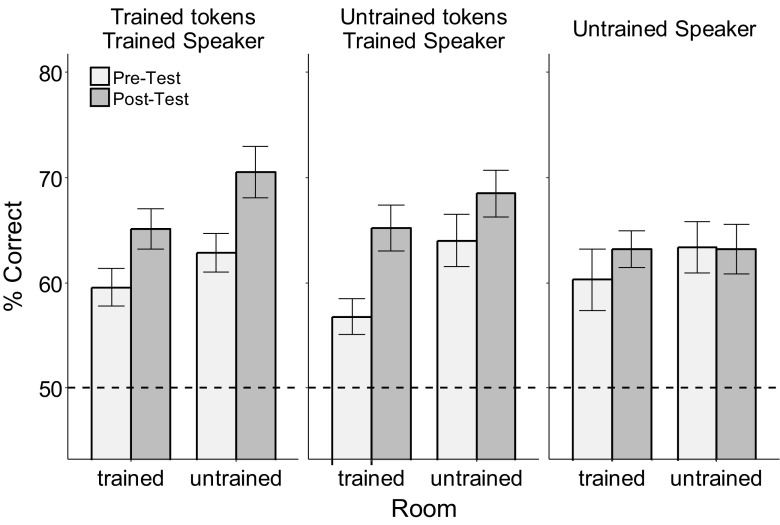
Performance for the three-room condition at pretest and posttest, averaged across the trained rooms (anechoic, bathroom, and classroom) and the untrained rooms (cafeteria and office) and plotted separately for the trained and untrained speakers and tokens. Dashed line indicates chance performance (50%)

To summarize the generalization of learning observed in this study, the 3R data were reanalyzed with the trained speaker’s trained and untrained tokens treated separately and with the rooms grouped by whether they were used in the training or not. Figure 4 shows pretesting and posttesting performance for the trained and untrained speakers and speech tokens as a function of the room group. The trained rooms group represents the average of the anechoic, bathroom, and classroom data, and the untrained rooms group represents the average of office and cafeteria data. Confirming the previous results, for the trained speaker (left-most and middle column in Fig. 4), participants improved approximately equally across trained and untrained speech tokens and rooms. For the untrained speaker (right-most panel), there was no evidence of learning in either room type. Thus, the implicit training in this study generalizes to untrained tokens of the trained speaker and to the untrained rooms, but not to untrained speakers. These results were confirmed by a repeated-measures ANOVA, with time, room group (trained, untrained), and token type (trained tokens, untrained tokens, untrained speaker) as factors, which found significant main effects of time, *F*(1, 14) = 11.38, *p* = .005, η_p_^2^ = 0.45, and room group, *F*(1, 14) = 10.21, *p* = .007, η_p_^2^ = 0.42, as well as significant interactions of Time × Token Type, *F*(2, 28) = 4.42, *p* = .028, η_p_^2^ = 0.24, while there were no interactions involving time and room group: Room Group × Time, *F* < 1; Room Group × Token Type × Time, *F*(2, 28) = 1.59, *p* = .22.

### Training

**Fig. 5:**
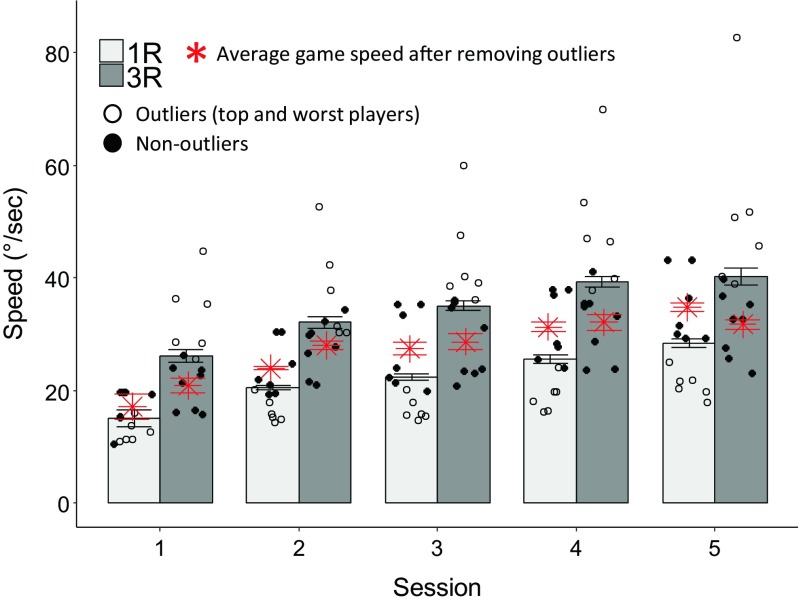
Average speed (°/sec) as a function of training session for 1R and 3R conditions, collapsed across rooms

We analyzed performance during training to test whether it could explain the differences in phonetic learning between the single room and multiple room conditions. The training game speed was evaluated here as an indirect measure of performance. Bars in Fig. 5 shows average speed over the first–fifth sessions, for the 1R and 3R conditions, whereas circles show individual participant data. Participants in the three-room training group were consistently faster in the game, but both groups showed similar improvement in performance (increase in the game speed) across each session. A mixed ANOVA, with condition (1R, 3R) as a between-participants factor and session (1–5) as a within-participants factor, found a main effect of condition, *F*(1, 25) = 13.30, *p* = .002, η_p_^2^ = 0.35, confirming that participants in the 3R condition were faster than those in 1R. It also found a main effect of session, *F*(4, 100) = 56.01, *p* < .001, η_p_^2^ = 0.69, suggesting that, overall, participants became faster in the game during training. However, importantly, there was no interaction (*F* < 1), suggesting that both groups improved equally during implicit training.

The observed faster performance in the three-room condition could be a potential confound. For example, it could mean that the 3R players were, on average, more experienced videogame players, which might have enhanced their abilities on a wide variety of perceptual tasks (e.g., Bejjanki et al. 2014; Green, Li, & Bavelier, 2010) and allowed them to benefit more from implicit training. To examine this further, we analyzed pretesting and posttesting performance after removing the six best performers from the 1R and the six worst performers from the 3R conditions, respectively, based on overall speed in the game, collapsed across sessions (outliers indicated by open circles in Fig. 5). The asterisks in Fig. 5 show average game speed after removing the best and worst performers from the 3R and 1R groups, respectively. The asterisks corresponding to the 1R and 3R groups are well aligned in each session, showing that performance in the game is similar across the groups. Importantly, the basic effect of 1R versus 3R training is unaffected by removing these participants (data not shown), producing results very similar to those shown in Fig. 5. Thus, it can be concluded that the better training performance (faster average on-screen character motion) in the 3R group is most likely due to a random imbalance between the participant groups, but that it does not confound the greater learning observed in the 3R group compared with the 1R group.

### Baseline data

**Fig. 6:**
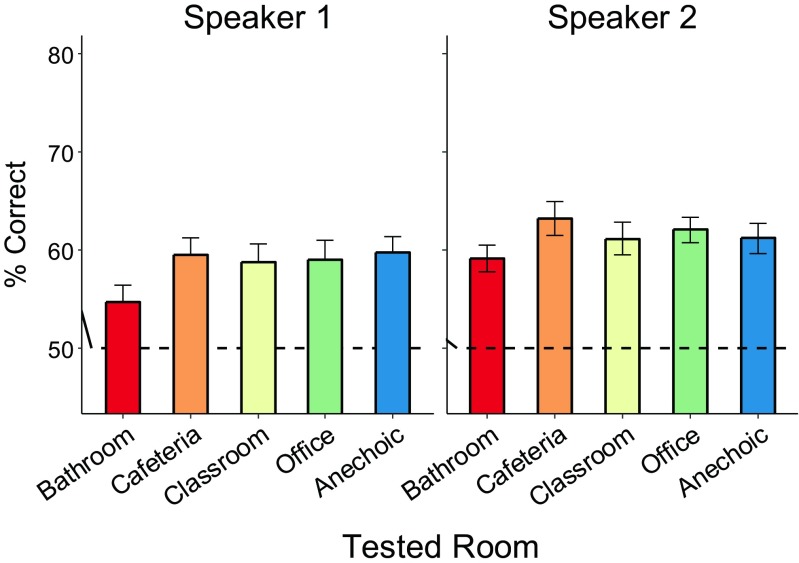
Pretest performance averaged across all participants and conditions, plotted as a function of testing room, separately for the two Hindi speakers. The dotted line indicates chance performance

To ensure that training effects are not explained by differences in baseline performance we examined baseline performance of participants to the phonetic stimuli across the different rooms with varying reverberation and the two speakers. Figure 6 shows pretest performance as a function of testing room, averaged across all 41 participants (i.e., averaged across conditions) and plotted separately for the two Hindi speakers (during training, only one of the two speakers was used for each participant, counterbalanced across participants). Accuracy was slightly higher for the second Hindi speaker, but performance was overall comparable across the two voices. Performance varied across rooms, such that for both speakers it was consistently worse for bathroom and similar for the other rooms. A two-way repeated-measures ANOVA with factors of Hindi speaker (Speaker 1, Speaker 2) and room (bathroom, cafeteria, classroom, anechoic, and office) confirmed this, showing a main effect of room, F(4, 160) = 3.14, *p* = .023, η_p_^2^ = 0.07, a trend for an effect of speaker, F(1, 40) = 3.23, *p* = .079, η_p_^2^ = 0.07, and no interaction between speaker and room (*F* < 1, *ns*).

These results suggest that nonnative listeners were initially able to better overcome distortions caused by reverberation in rooms with modest levels of reverberation (office, classroom, and cafeteria) than in the very reverberant bathroom. Specifically, averaged across the two Hindi speakers, performance in office, classroom, cafeteria, and anechoic room was almost constant at approximately 60.6%. This matched well the initial performance of 61.2% obtained without any room simulation in a previous study that used the same phonetic distinction from the same Hindi speakers (Vlahou et al., 2012). For the more challenging bathroom environment, performance dropped to 56.9%, consistent with acoustic analyses showing that bathroom had the largest T_60_ and lowest C_50_ out of the rooms examined here (see Table 2). This shows that, although the bathroom reverberation might not be overly disruptive for understanding conversational speech, in the context of a difficult phonetic identification task performed by nonnative listeners, it caused a significant decline in performance. Importantly, however, even the bathroom performance is still above chance, *t*(40) = 5.44, *p* < .0001), suggesting that participants were able to distinguish the phonetic contrast to some extent prior to training.

## Discussion

This study examined implicit learning of new phonetic categories by nonnative listeners when training was performed in fixed versus varying acoustic environments. Our main finding is that exposure to varying rooms during training can induce this type of learning, while exposure to a single, anechoic or reverberant, room showed no evidence of learning. The study also found that the learning induced by the three-room training using one specific speaker generalized to untrained tokens of the same speaker and to untrained rooms, while not generalizing to the untrained speaker. These findings have potential implications for our understanding of adult phonetic plasticity in complex listening environments, typical of everyday communication.

Several mechanisms might underlie the implicit learning that requires varying acoustic environments observed here. One possibility is that exposure to various rooms can promote the implicit extraction of acoustic features in speech stimuli that are robust against reverberation, resulting in identification of invariant features and formation of new phonetic categories. Previous work has shown that by increasing the variability along a previously preferred acoustic dimension, listeners might shift their perceptual weighting toward more reliable acoustic cues (Lim & Holt, 2011). In the current study, depending on the listening environment and the acoustic properties of the sound, some phonetic cues might become severely disrupted and unreliable, whereas others become more salient and stand out. Several previous studies showed that, depending on context, listeners can change which cues they use for a specific phonetic categorization. For example, Parikh and Loizou (2005) showed that native listeners perform well for stop consonants even when the stop’s burst cues became unreliable due to noise, suggesting that in adverse conditions the listeners rely on other cues, such as spectral change and formant transitions. Similarly, Mattys, Davis, Bradlow, and Scott (2012) showed that when listeners were asked to segment a short string of syllables, they relied more on coarticulatory cues in clear listening conditions, but more on stress in noisy conditions. In our study the listeners might be requiring the exposure to multiple rooms to identify the relevant phonetic cues and start implicitly learning and combining them.

A related issue is that in our study participants were exposed to a consistent room in each training block. Consistent room exposure has been shown to benefit speech perception for native listeners (Brandewie & Zahorik, 2010; Srinivasan & Zahorik, 2013), presumably allowing them to take advantage of regularities in the distortions that the reverberation pattern specific to each room imposes upon the exposed phonemes. The current results suggest that a more detailed mapping between phonetic cues and room acoustics could be obtained in varying acoustic environments, resulting in richer and more elaborate perceptual representations, upon which reinforcing signals can act. If that is the case, then varying rooms on a trial-by-trial basis might result in an even stronger implicit learning.

The finding of weaker transfer of learning to an untrained voice is in line with the well-known observation in the phonetic learning literature that talker variability during training is crucial for generalization of learning to new voices (Lively et al., 1993; Logan et al., 1991; Pruitt et al., 2006). Logan et al. (1991) trained Japanese listeners with words that contained /r/ and /l/ in various syllable positions produced by five different speakers. Following this high-variability training regime, participants showed robust learning of the training set and transfer to novel tokens and voices. Lively et al. (1993) further demonstrated that listeners trained with a broad range of stimuli produced by a single talker failed to show transfer of learning to new voices, whereas participants trained with multiple voices were able to overcome phonetically irrelevant, talker-specific representations and showed learning for novel words produced by novel voices. More recently, Pruitt et al. (2006) used an adaptive training procedure in which more difficult phonetic stimuli and additional speakers were gradually introduced, and showed robust transfer of learning across multiple dimensions simultaneously, including both vowel and consonant contexts and a new voice. These studies further substantiate that variability during training along critical acoustic-phonetic dimensions supports transfer of learning across these dimensions. In the present study, our primary focus was on the effects of varying room environments on phonetic learning, therefore performance on the untrained speaker was not examined further. However, it would be interesting to investigate how varying voices would interact with varying acoustic environments during implicit training.

At first glance, the finding of no learning for 1R training is surprising, and seemingly contradictory to prior results in which implicit learning has been found on these same Hindi stimuli without manipulation of room reverberation (Vlahou et al., 2012). Although there are a number of possible explanations that may reconcile these findings, an important difference is likely that the implicit training approach used in our study differed from the one used in the prior study in that the phonetic tokens were not predictive of targets in the videogame. In our previous work, the sounds from one category always predicted the task target, thus learning the sound differences (even without a clear awareness of doing so) could have benefitted participants (Vlahou et al., 2012; although see Seitz & Watanabe, 2008). In contrast, here, sounds from one category were only played after the participants successfully responded to the target. Although the results of the multiple room training showed that this is indeed sufficient to result in learning, the timing of the sound presentation may be suboptimal, and other factors that normally contribute to implicit learning (such as direction of attention or predictiveness of the sounds) may be lacking, resulting in poorer overall learning. In this case, it may be that the extra stimulation found through presenting diverse acoustic environments (3R condition) was needed to boost activity above a learning threshold, as proposed by Seitz and Dinse (2007).

Although this study provides an important first step in addressing the effects of reverberation on nonnative phonetic learning, there are several limitations that need to be pointed out. First, although there is evidence that the current implicit training paradigm failed to induce learning when training was performed in a fixed—anechoic or reverberant—room, the analysis of 1R subgroups was underpowered, thus preventing us from drawing firm conclusions as to whether the primary cause was the lack of reverberation or room variation. Second, even though we used acoustic environments that are arguably typical of everyday listening, the range of tested reverberation times was limited. Future work can investigate whether similar results would be obtained using additional rooms, including environments with more severe reverberation and noise. Furthermore, the stimulus set included two speakers, with only one speaker used during training, thereby lacking the necessary information that would support generalization of learning to new voices (e.g., Lively et al., 1993). Also, the effects of reverberation were examined using only one phonetic contrast. Thus, it is unclear whether these results would generalize beyond the specific sounds, to cover a wider range of nonnative phonetic categories. Finally, as noted above, the particular implicit training variant that we used might be suboptimal, diminishing learning effects for the more severe reverberant environments. Thus, though these results provide a first insight into the effects of varying acoustic environments on phonetic learning, future work needs to expand our findings and refine our understanding on nonnative phonetic learning in complex listening environments.

In summary, investigating phonetic learning in situations that resemble more real-world settings is important to help us understand adult phonetic plasticity. So far, no studies have investigated how exposure to a varying room environment affects the acquisition of novel phonetic categories. Even modest levels of reverberation can degrade the speech signal; still, normal-hearing native listeners rapidly and flexibly adjust cue weighting and compensate for interfering effects of room acoustics. Thus, perceptual adaptation mechanisms have been postulated that take into account contextual reverberation patterns (Brandewie & Zahorik, 2010; Creel et al., 2012). Here, we showed that nonnative listeners also achieve improved performance in reverberation and even benefit from experiencing the novel phonemes in different rooms during implicit training. Further, it is notable that the current results are not consistent with the view that adult plasticity is restricted to stimuli that predict behaviorally relevant events (e.g., Polley et al., 2008; Polley, Steinberg, & Merzenich, 2006). Altogether, these results have potential implications for the design of effective, ecologically inspired phonetic training applications, suggesting that rather than disrupting the learning process, exposure to multiple rooms can enhance unsupervised phonetic learning.
